# Recent Developments on Therapeutic and Diagnostic Approaches for COVID-19

**DOI:** 10.1208/s12248-020-00532-2

**Published:** 2021-01-05

**Authors:** Joydeb Majumder, Tamara Minko

**Affiliations:** 1 Department of Pharmaceutics, Ernest Mario School of Pharmacy, Rutgers, the State University of New Jersey, 160 Frelinghuysen Road, Piscataway, New Jersey 08854 USA; 2 Rutgers Cancer Institute of New Jersey, New Brunswick, New Jersey 08903 USA; 3 Environmental and Occupational Health Science Institute, Piscataway, New Jersey 08854 USA

**Keywords:** antiviral drugs, anti-SARS-CoV-2 antibody, antiviral vaccines, ARDS, convalescent plasma therapy, immunotherapy, nanotherapeutics

## Abstract

The ongoing pandemic of coronavirus disease 2019 (COVID-19) caused by the severe acute respiratory syndrome coronavirus 2 (SARS-CoV-2) has made a serious public health threat worldwide with millions of people at risk in a growing number of countries. Though there are no clinically approved antiviral drugs and vaccines for COVID-19, attempts are ongoing for clinical trials of several known antiviral drugs, their combination, as well as development of vaccines in patients with confirmed COVID-19. This review focuses on the latest approaches to diagnostics and therapy of COVID-19. We have summarized recent progress on the conventional therapeutics such as antiviral drugs, vaccines, anti-SARS-CoV-2 antibody treatments, and convalescent plasma therapy which are currently under extensive research and clinical trials for the treatment of COVID-19. The developments of nanoparticle-based therapeutic and diagnostic approaches have been also discussed for COVID-19. We have assessed recent literature data on this topic and made a summary of current development and future perspectives.

## INTRODUCTION

Newly emerging virus diseases have become a major public health threat around the world in recent years. During the last two decades, outbreaks of several viral diseases have been reported including the severe acute respiratory syndrome coronavirus (SARS-CoV) (1) in 2002, H1N1 influenza (2) in 2009, the Middle East respiratory syndrome coronavirus (MERS-CoV) (3) in 2012, Ebola virus disease (EVD) (4) in 2013, and Zika virus (5) in 2015. The most recent and ongoing viral disease caused by the novel coronavirus has severely threatened public health worldwide. The outbreak of novel coronavirus was initially reported to the World Health Organization (WHO) on December 31, 2019. On January 12, 2020, WHO designated this virus as novel coronavirus “2019-nCoV” (6), and later, it was termed as the severe acute respiratory syndrome coronavirus-2 (SARS-CoV-2) by the International Committee on Taxonomy of Viruses (ICTV) because of its similarity with the previous SARS-CoV (7). On January 30, 2020, WHO declared this viral outbreak as a public health emergency of international concern (8). Later on February 11, 2020, WHO termed this disease as coronavirus disease 2019 (COVID-19) (9) and, subsequently on March 11, 2020, WHO declared COVID-19 as a global pandemic since the SARS-CoV-2 viral infection has spread rapidly in a growing number of countries (10). As of October 31, 2020, more than 45 million people were infected with the COVID-19 disease, and 1.2 million deaths have been reported globally (11). While human-to-human transmission rate is higher for SARS-CoV-2 than that of SARS-CoV virus, the mortality rate of COVID-19 disease is much lower than that of SARS-CoV infection (12). While all coronaviruses distress the respiratory tract, SARS-CoV-2 virus additionally also affects the heart, gastrointestinal system, liver, kidney, and the central nervous system eventually leading to multi-organ failure (13,14).

**Fig. 1:**
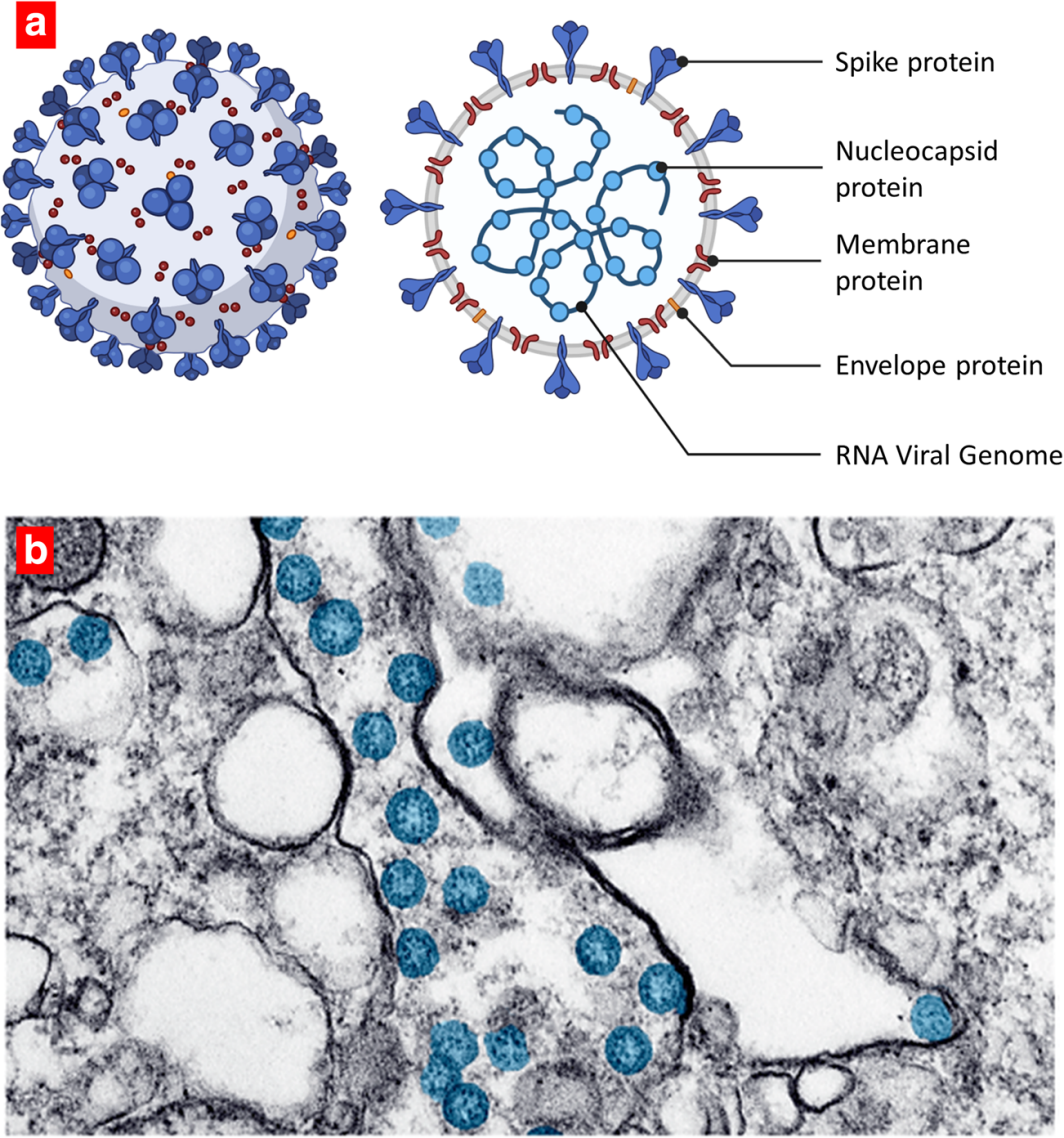
Human coronavirus. **a** Schematic structure of SARS-CoV-2 virus. **b** A transmission electron microscope image of SARS-CoV-2 spherical viral particles in cell. The virus is colorized in blue. Adapted from the US Centers for Disease Control. Reproduced with permission from (16)

SARS-CoV-2 genome contains single-stranded positive-sense RNA encapsulated within a membrane envelop with an average diameter of 75–150 nm. The envelope is covered with glycoprotein spikes giving coronaviruses their crown-like appearance (corona is the Latin term for crown or garland). The genome of SARS-CoV-2 has a length of about 30 K nucleotides. Almost 85% homology of this virus is also similar to the SARS-CoV (15). Four main structural proteins are encoded in SARS-CoV-2 viral genome, namely, spike (S) surface glycoprotein, membrane (M) protein, envelope (E) protein, and nucleocapsid (N) protein (Fig. 1a). Several other nonstructural proteins are also encoded by the viral genome (RNA polymerase, RdRp; papain-like protease, PLpro; coronavirus main protease, 3CLpro) (17). A representative transmission electron microscope (TEM) image of SARS-CoV-2 viruses in host cells is shown in Fig. 1b, where the SARS-CoV-2 viruses have been colorized in blue color (16).

**Fig. 2:**
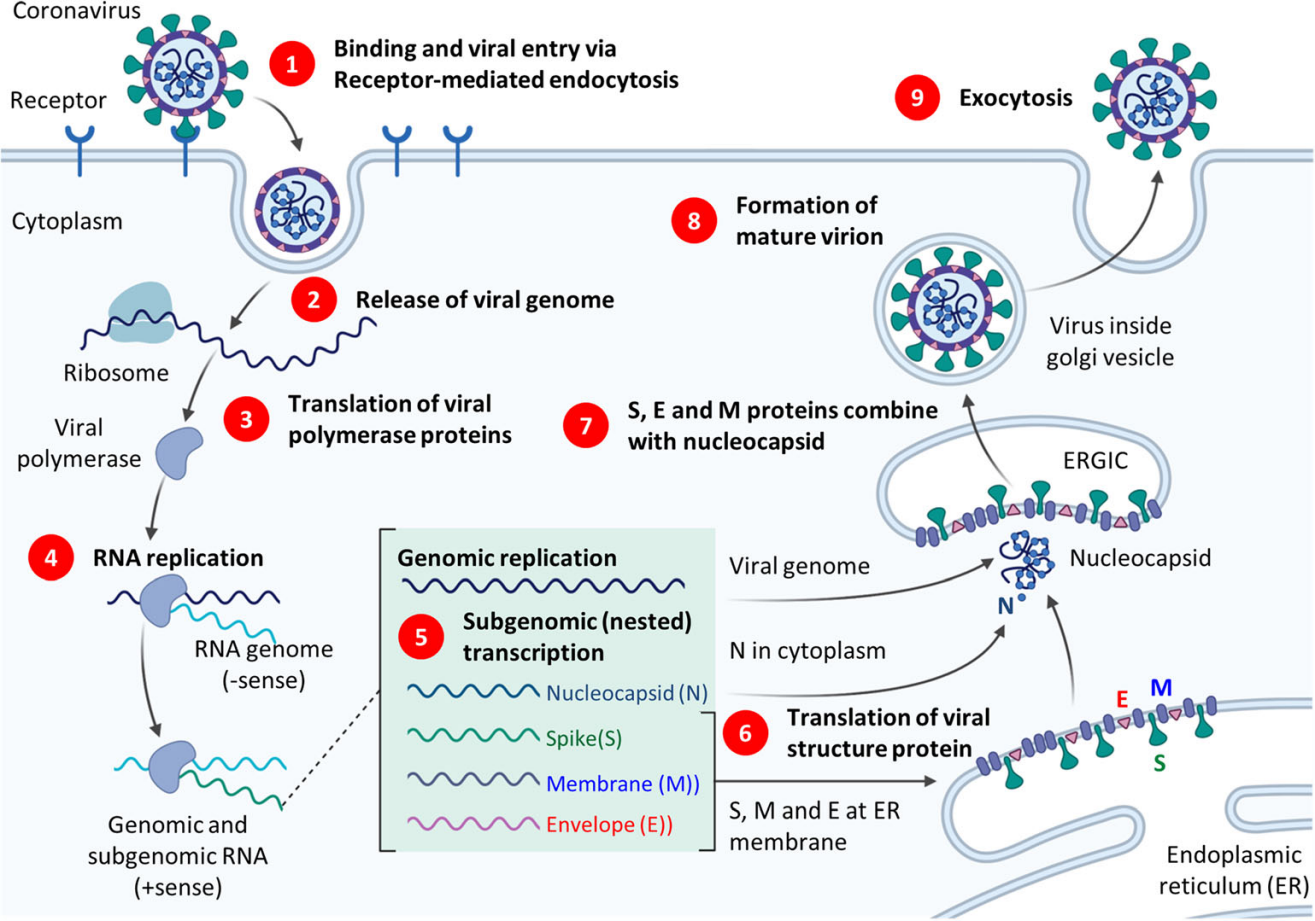
Overview of the coronavirus replication cycle (simplified, not to scale). The figure depicts viral development from initial binding and release of viral genome to eventual exocytosis of the mature virion. ERGIC, endoplasmic reticulum-Golgi intermediate compartment (also referred to as vesicular-tubular cluster). Drawn using a template retrieved from (18)

The replication of COVID-19 as any other virus requires a host cell and normally includes attachment, penetration, uncoating, replication, assembly, and release steps. A schematic representation of cellular uptake and replication of SARS-CoV-2 virus is shown in Fig. 2 (18). Spike glycoprotein on the surface of the COVID-19 binds to angiotensin-converting enzyme 2 (ACE2) receptor protein located on the host cell plasma membrane and facilitates the host cell invasion. Serine protease TMPRSS211 produced by a host cell facilitates the process of this invasion. After receptor-mediated endocytosis of the virus into the host cells, it releases viral genome (single-stranded positive RNA), and using host ribosome translates into viral polyproteins. Viral proteinases 3CLpro and PLpro cleave viral polyproteins into effector proteins. RNA-dependent RNA polymerase in turn synthesizes a full-length negative-strand RNA template which is used to make more viral genomic RNA. Viral genome then is synthesized by genomic replication, and four essential structural viral proteins (N, S, M, and E) are produced by transcription then translation. N protein binds genomic RNA while S, M, and E proteins are integrated into the membrane of the endoplasmic reticulum (ER) forming ERGIC—endoplasmic reticulum-Golgi intermediate compartment (also referred to as vesicular-tubular cluster). Assembled nucleocapsid with helical twisted RNA is encapsulated into the ER lumen, viral progeny is transported by the ERGIC toward the plasma membrane of the host cell, and finally, daughter virus is released by exocytosis.

In general, antiviral treatment and prevention approaches are based on the (a) inhibition of the replication of the viral genome by either preventing virus entering host cells or suppression of one or more steps in its replication, (b) priming the immune system and creating a form of memory against the virus by the vaccination, (c) injection of the antiviral antibodies produced in the plasma from the recovered patient to the infected patient, and (d) treatment of lung damage and respiratory distress syndrome accompanied the viral infection. In this review, we have summarized the ongoing developments of therapeutic and diagnostic approaches based on the conventional methodologies for COVID-19. Besides the conventional methods, we have presented recent developments of various nanocarrier-based therapeutic and diagnostic approaches for COVID-19.

Coronavirus disease 2019 (COVID-19) is defined as a disease caused by the severe acute respiratory syndrome coronavirus 2 (SARS-CoV-2). The signs and symptoms of COVID-19 disease are different from patient to patient, but most common clinical symptoms include fever, fatigue, cough, expectoration, anorexia, sputum production, shortness of breath, etc. during various stages of this disease (19,20). Besides, less common symptoms such as sore throat, headache, confusion, hemoptysis, shortness of breath, and chest tightness have been also observed (21,22) as well as minor symptoms such as nausea, vomiting, diarrhea, and gastrointestinal complication were also reported (23). Similar signs and symptoms of COVID-19 were observed in children like the adults, but those symptoms were usually milder as compared with the adult patients (24,25). However, there were patients not yet symptomatic (presymptomatic) as well as there were patients (asymptomatic) with no typical symptoms of COVID-19 disease as revealed in many reports (26–29). Several asymptomatically infected COVID-19 patients were found to be asymptomatic throughout the period of the infection (30,31). A recent study also revealed that the rate of human-to-human transmission of SARS-CoV-2 virus is higher than that of the previous two coronaviruses SARS-CoV and MERS-CoV infections.

To date, there is no effective therapy for COVID-19. Therefore, the key management of COVID-19 patients included early diagnosis, immediate patient isolation, and protective conditions to prevent the infection. Typical treatment for COVID-19 disease included general supportive care, respiratory support, as well as nutritional support.

## COVID-19: THERAPEUTIC APPROACHES

Currently, there are no clinically approved drugs or therapeutics by the U.S. Food and Drug Administration (FDA) for the treatment of COVID-19. Traditional drug discovery approaches are time-consuming, and trial-and-error methods are often ineffective. Because of regulatory requirements to assess safety and efficacy of drug, the time required to develop a new drug takes average one to two decades. A rational approach to overcome the sustained high failure rates and the time and costs involved in the research and development to bring a new drug in the market is to repurpose existing drugs based on similarity of disease mechanisms for the targeted drugs (32). Moreover, repurposed drugs have the advantage of less development costs and time because of their previously available pharmacokinetic, toxicology, and safety data. Taking repurposing as a primary strategy, many randomized controlled trials of known drugs are ongoing in patients with COVID-19 (33–35). Recently, both researchers and clinicians have been paid significant attention for developing vaccines and antiviral therapeutics. On August 2020, more than 350 COVID-19 therapeutic drugs (mostly repurposed) are investigated with more than 75% of them entered in human clinical trials, and more than 150 repurposed and original vaccines are under study with about 25% of them on the various phases of human trials (36).

### Antiviral Drugs

#### Treatment Mechanisms

**Fig. 3:**
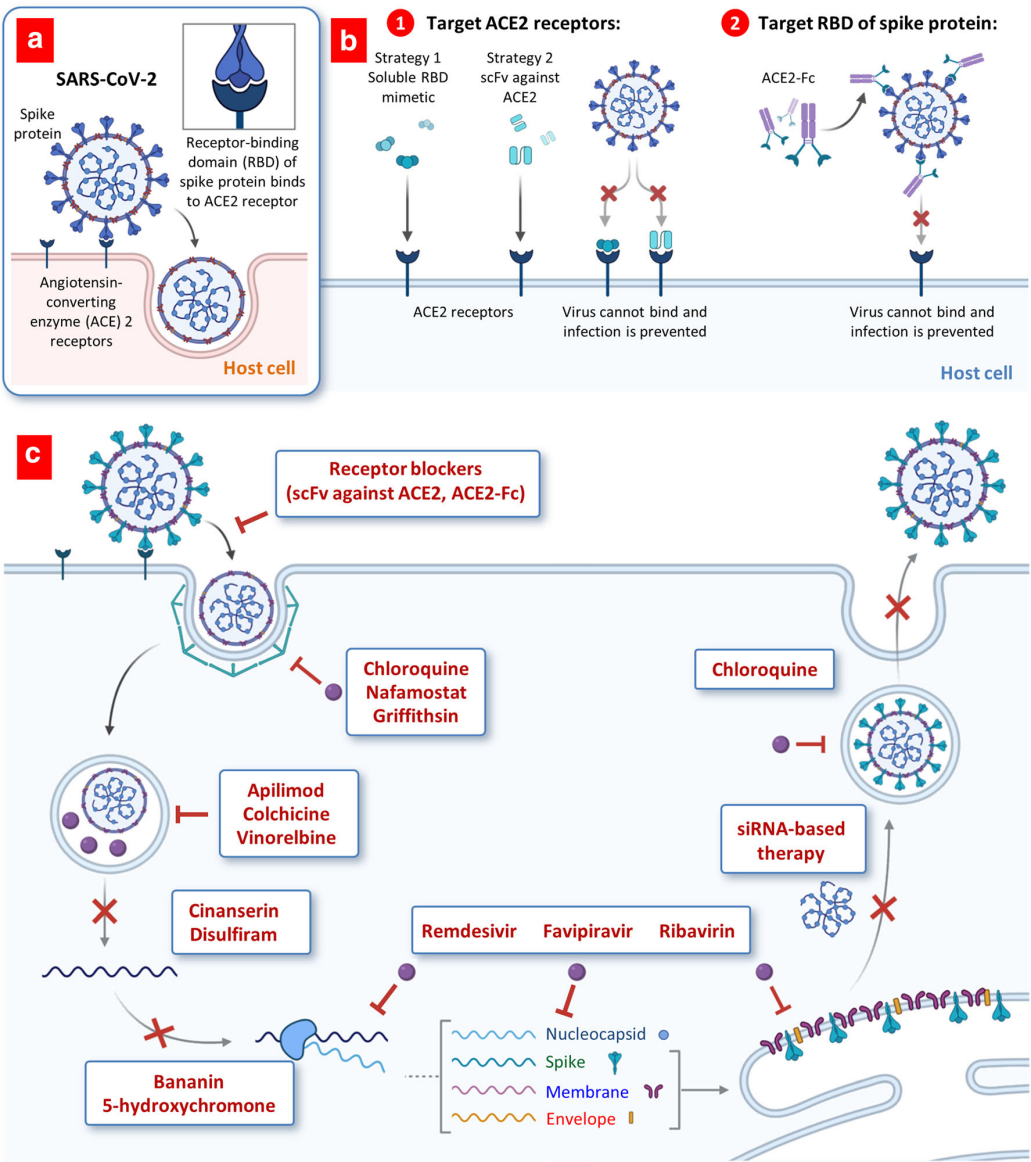
Proposed therapeutic treatments for COVID-19. **a**, **b** Targeting viral entry mechanism. **a** Viral entry mechanism of SARS-CoV-2. **b** Possible approaches for blocking ACE2 receptors. **c** Antiviral drugs targeting the coronavirus replication cycle (simplified). Abbreviations: ACE2, angiotensin-converting enzyme 2; scFvs, recombinant human single-chain variable region fragments against the S1 domain of spike (S) protein of the SARS-CoV; ACE2-Fc, immunoglobulin fragment (Fc)-ACE2 fusion protein

In general, two distinct approaches are being explored for repurposing of conventional drugs and development of novel therapeutic drugs: prevention of virus entry into the host cells and suppression of various steps in virus replication inside the cells (Fig. 3).

#### Blocking Viral Entry

COVID-19 viruses target angiotensin-converting enzyme (ACE) 2 receptors overexpressed in the cells of lung and gastrointestinal tissues in the human body (Fig. 3a). The receptor-binding domain (RBD) of spike protein on the surface of the coronavirus binds to ACE2 on the plasma membrane of infected cells, initiating receptor-mediated endocytosis. Three strategies are currently investigating in order to block this interaction and abrogate infection (37). First, a high number of soluble RBD mimetics can be administered to bind ACE2 receptors and saturate available sites, preventing virus binding and entering host cells (Fig. 3b (1)). Second, antibodies or single-chain antibody fragments (scFv) against ACE2 receptors can also be administered to accomplish a similar task. Third strategy targets the coronavirus RBDs directly by using the ACE2 extracellular domain in order to bind to the spike protein. A fragment crystallizable (Fc) region (the tail fragment of an antibody) that interacts with ACE2 cell surface receptors bonded directly to RBDs of the virus again can prevent its interaction with the host cells and preventing infection. Despite extensive research for developing various antiviral therapeutics that are capable to prevent or at least limit binding of SARS-CoV-2 virus to ACE2 receptors, certain aspects of these approaches may potentially limit their efficacy. First, native mimetics, antibody, and their fragments possess a relatively low stability in the blood stream and may drop their specific activity during their transfer to their primary targets (ACE2 receptors in lung cells and RBDs in the virus) (38). On our opinion, the most effective ways to protect therapeutic payload from the degradation in the blood stream include their conjugation to nanoparticles and delivery specifically to the site of action. In the case of COVID-19 and other SARS viruses, inhalation delivery of ACE2 antibodies by nanoparticles specifically to the lungs is capable not only to deliver them as close as possible to the targeted cellular receptors and preserve their specific activity but also substantially limit the inactivation of ACE2 receptors in other non-targeted organs limiting possible adverse side effects of the treatment. Currently, we are carrying out the experiments for delivering of ACE2-targeted antibodies using lipid nanoparticles by inhalation. Other limitations include possible binding of remedies to sites on the ACE2 receptors or RBDs that do not compete or interfere with viral entry. The potency of such therapeutics can be improved by enhancing their binding affinity against the epitope of the RBD (39).

#### Targeting the Coronavirus Replication

In contrast to ACE2 receptor blockers, the most part of repurposed anti-COVID-19 drugs do not interact with the viral entry to the host cells. Instead, drugs block one or several steps of virus replication inside the cell (Fig. 3c). Drugs may prevent endocytosis (e.g., chloroquine, nafamostat, griffithsin), inhibit endosome maturation (e.g., hydroxychloroquine, apilimod, colchicine, vinorelbine), and release of viral genome (e.g., cinanserin, disulfiram) and virus replication, transcription, and translation of viral proteins (e.g., bananin, 5-hydroxychromone, remdesivir, favipiravir, ribavirin). siRNA-based therapeutics and some repurposing drugs can also be employed to prevent the formation of daughter virion and its release from the host cell by exocytosis. A summary of various repurposed drugs in clinical trials for treatment of COVID-19 with original application, targeted molecules, and brief description of a major mechanism of action with references are shown in Table I. Below, we provide brief information of several repurposed and original antiviral drugs, antibodies, and vaccines proposed and tested for the treatment of COVID-19 infection.

**Table I:** List of Drugs Repurposed in Clinical Trials for COVID-19. Modified From (40)

Drugs	Applications	Targets	Mechanism of action against life cycle of SARS-CoV-2 virus	References
Favipiravir	Viral diseases	RNA-dependent RNA polymerase (RdRp)	Inhibits viral RNA polymerase and RNA replicase activity	(41)
Remdesivir	Ebola virus diseases (EVD)	Viral proteases, RdRp	Interferes with viral RNA polymerase activity and reduces viral RNA synthesis to arrest viral replication	(8,42)
Lopinavir	HIV infections	Viral proteases	Inhibits the viral proteases activity	(9,10)
Ritonavir	HIV infections	Viral proteases	Inhibits the viral proteases activity	(9,10)
Chloroquine	Malarial	Angiotensin Converting Enzyme-2 (ACE2)	Interferes with glycosylation of ACE2	(43,44)
Ribavirin	Respiratory syncytial virus (RSV) infection	RdRp	Inhibits viral RNA polymerase activity	(45)
Umifenovir (Arbidol)	Influenza	S-spike glycoprotein	Inhibits membrane fusion and prevent viral entry into the host cells	(46)

#### Remdesivir

Remdesivir, a recently discovered novel antiviral drug in the class of nucleotide analogs, has shown potent effect in treating Ebola virus and Marburg virus infections (47). Remdesivir was developed by Gilead Sciences for the treatment of viral diseases caused by various RNA viruses. The drug displayed antiviral activity against various single-stranded RNA viruses such as Nipah virus, Hendra virus, and coronaviruses (including MERS and SARS-CoV viruses) (48,49). More recently, remdesivir is being studied for the treatment of COVID-19 infections. On May 1, 2020, the US-FDA approved an emergency use authorization (EUA) for remdesivir based on the preliminary clinical data indicating quick recovery of the COVID-19 patients (50). In a recent clinical trial study, patients with confirmed COVID-19 infection were treated with compassionate use of remdesivir for 10 days (51). On day 1, patients were treated intravenously with 200 mg dose. Then, treatment with 100-mg intravenous daily dose was continued for another 9 days. The results showed clinical improvements for 36 of 53 patients (ca. 68%). In a recent study, Spinner *et al.* (52) conducted a randomized and open-label phase 3 clinical trial of remdesivir in total 584 patients with moderate COVID-19 disease (ClinicalTrials.gov Identifier: NCT04292730). All the patients were randomized in three groups: 197 patients in 10-day course of remdesivir treatment, 199 patients in 5-day course of remdesivir treatment, and 200 patients in standard care treatment only. Remdesivir was injected intravenously at a dose of 200 mg on day 1 followed by daily treatment of 100 mg for an entire treatment course. Initial results revealed that clinical status distribution was better in the group of patients randomized to a 5-day course of remdesivir treatment when compared with that of the standard care group. More recently, the FDA has approved the antiviral drug Veklury (remdesivir) which inhibits replication of SARS-CoV-2 virus for the treatment of patients with COVID-19 requiring hospitalization (53).

#### Umifenovir

Umifenovir is an indole-based antiviral agent, which is mainly used for the treatment of influenza and other respiratory diseases in Russia and China (54). Umifenovir was found to show activity against other type of RNA and DNA viruses such as Zika virus, Lassa virus, and Ebola virus (55,56) Antiviral activity of umifenovir may be attributed to interactions of its aromatic residues with the viral glycoproteins, which is involved in viral fusion through the cell membrane (57,58). Currently, this drug is being investigated for the treatment of COVID-19 infection (59,60). In an early report, Lian *et al.* performed a retrospective study of umifenovir in 81 COVID-19 patients. All the patients were divided into control group (*n* = 36) and umifenovir treatment group (*n* = 45). After 7 days of post-treatment, no deaths were reported in both groups; however, 28 out of 36 (78%) patients in the control group tested negative for SARS-CoV-2 infection, whereas similar numbers (33 out of 45 or 73% patients) were obtained in the umifenovir treatment group. Thus, the initial results revealed that umifenovir treatment did not improve outcomes in COVID-19 patients (61). Recently, Chen *et al.* performed a retrospective cohort study in 200 patients with COVID-19 to see the effect of umifenovir (Arbidol) in combination with Shufeng Jiedu Capsules for 2 weeks. The control group patients were treated with Arbidol hydrochloride capsules, and the experimental group patients were treated with combination of Arbidol hydrochloride capsules and Shufeng Jiedu Capsules. The subsidence of a fever was observed more rapidly in the experimental group of participants. The chest CT scan also showed better resolution of pneumonia symptoms in the experimental group than that in the control group (62).

#### Favipiravir

Favipiravir is a pyrazine class antiviral drug, which was mainly used for the treatment of influenza in Japan (63,64). It works by inhibiting the function of RNA-dependent RNA polymerase (RdRp) enzymes, the protein which is involved in the transcription and replication of viral genomes (65). Besides influenza, it was also investigated for the treatment of Ebola virus, and recently, it has been evaluated for the treatment of COVID-19 (66).

#### Chloroquine

Chloroquine is an aminoquinoline class small molecule drug, which is used for the treatment of malaria. Chloroquine was also repurposed for the treatment of other diseases such as HIV and rheumatoid arthritis (67). This drug potentially can have a wide spectrum of antiviral action on all steps of the viral entry and replication (68). Latest studies revealed that chloroquine inhibits the process of glycosylation of angiotensin-converting enzyme 2 (ACE2), an enzyme which is present on the cell membranes of cells in the lungs, arteries, heart, kidney, etc. and found to involve in the mechanism of cellular entry of SARS-CoV and SARS-CoV-2 viruses (69,70). Recently, the FDA has issued an emergency use authorization for chloroquine for the treatment of COVID-19 (71).

#### Clinical Trials of Antiviral Drugs

Currently, there are a number of drug candidates in various stages of clinical trials for the treatment of COVID-19 (72,73). On the end of September 2020, 322 out of total 409 drug candidates are tested in human trials, with 119 of them entered phase III and 46—phase IV (36). Here, we have summarized results from few clinical trials of antiviral therapeutics for COVID-19. Remdesivir is a broad-spectrum antiviral agent which works by inhibiting the viral RNA-dependent RNA polymerase activity. Remdesivir has been found to show activity against the previous SARS-CoV and MERS-CoV infections (74). Recently, Beigel *et al.* conducted intravenous treatment of remdesivir in a clinical trial of total 1063 adult patients with COVID-19 disease (42). All patients were randomly divided into two groups, namely, remdesivir and placebo groups. Patients in remdesivir group received 200 mg dose on day 1, followed by 100 mg daily dose for additional 9 days. Preliminary results revealed that patients of remdesivir group had average recovery time of 11 days as compared with 15 days of the patients who received placebo. Thus, remdesivir treatment demonstrated better clinical benefit than placebo in shortening the recovery time of adult patients with COVID-19. Another randomized clinical trial also revealed that remdesivir treatment reduced the recovery time from the SARS-CoV-2 infection (75). On May 1, 2020, the US Food and Drug Administration (FDA) issued an Emergency Use Authorization of remdesivir for the treatment of patients with severe condition of COVID-19. Currently, remdesivir is under clinical trial at the phase III level for the treatment of COVID-19 (76). Further clinical studies of remdesivir are ongoing to evaluate its safety and efficacy in more patients with SARS-CoV-2 infection. Favipiravir—an antiviral drug used to treat influenza—was investigated in a phase II clinical trial in China at the early days of the outbreak of SARS-CoV-2 infection for its efficacy and safety in COVID-19 patients (77). Later, favipiravir in combination with other antiviral drugs were also investigated in clinical trials for COVID-19. However, no significant therapeutic outcome was observed from these clinical studies of favipiravir for COVID-19 (Chinese Clinical Trial Registry Identifiers: ChiCTR2000030987, ChiCTR2000029600). Lopinavir/ritonavir is an FDA-approved combination agent for HIV treatment. Lopinavir/ritonavir combination agent is currently being studied in clinical trial for COVID-19 (78). Recently, Cao *et al.* performed a randomized controlled trial of lopinavir/ritonavir in 199 patients with COVID-19 disease (79). The efficacy of lopinavir/ritonavir treatment was compared with the standard care and management for COVID-19 patients. However, no significant difference was observed in terms of viral clearance or mortality rates in both the treated and standard care group of patients. Chloroquine, an antimalarial drug was also evaluated in clinical trials for COVID-19 (43). In March 2020, Gautret and co-authors performed an open-label randomized controlled trial of hydroxychloroquine (HCQ) in COVID-19 patients (44). All the treated patients received 200 mg of HCQ for 10 days with 3 doses per day, while the control group received usual care for COVID-19. Results revealed that, at day 6 post-inclusion, 70% of hydroxychloroquine-treated patients were virologically cured (had negative PCR results in nasopharyngeal samples) compared with 12.5% in the control group (*p* = 0.001). Other antiviral drugs such as ribavirin were also investigated in a randomized controlled trial in COVID-19 patients, but the results showed mixed success (45). More recently, glucocorticoids as dexamethasone were investigated in clinical trial of patients with severe COVID-19 (80). Initial results revealed that dexamethasone treatment reduced death rate of the COVID-19 patients by one-third as compared with the COVID-19 patients who received only standard supportive care treatment.

While promising preliminary results were observed from few of the ongoing clinical trials, still many concerns related to safety and toxicity of the repurposed drugs limited their further clinical studies. For example, based on the results of recent clinical trials, the US FDA revoked its Emergency Use Authorization (EUA) for both chloroquine and hydroxychloroquine (HCQ) for the treatment of COVID-19 (81). Recently, Risambaf *et al.* raised various concerns related to side effects of both chloroquine and hydroxychloroquine for the treatment of COVID-19 (82). Recent report also revealed that long-time use of chloroquine and hydroxychloroquine developed various toxicity effects including renal failure, cardiotoxicity, autoimmune disorders, etc. (83). The latest study of hydroxychloroquine in combination with azithromycin also did not show clinical benefits to the patients of COVID-19 (84). Therefore, more randomized controlled trials with higher number of participants are needed for further evaluation of chloroquine and hydroxychloroquine for the treatment of COVID-19.

### Monoclonal Antibodies

Monoclonal antibodies specific to a viral protein are alternative treatment options for viral disease. During the last decade, several targeted monoclonal antibodies were developed for the SARS-CoV spike protein to inhibit the viral fusion inside the host cells (85,86). For example, CR3014 and CR3022 are two SARS-CoV neutralizing monoclonal antibodies, which bind to the receptor-binding domain (RBD) of the SARS-CoV spike protein to neutralize the virus (87). Among these, CR3022 was also found to bind with the SARS-CoV-2 RBD (88), and it might be a possible therapeutic option for the treatment of COVID-19 disease. Recently, researchers have cloned two human anti-SARS-CoV-2 RBD-hACE2 blocking monoclonal antibodies (mAbs) from SARS-CoV-2 RBD-specific memory B cells of recovered COVID-19 patient (19). The authors found that these two mAbs are capable of blocking the interaction between the ACE2 receptor and SARS-CoV-2 RBD leading to neutralization of the SARS-CoV-2 spike protein. Such mAbs could be promising therapeutic agents against the SARS-CoV-2 infection. Molecular imprinting is a potent modern technology for the development of so-called monoclonal-type plastic antibodies (MTPA) based on molecularly imprinted polymers (MIPs). These polymeric materials possess specific recognition properties for target molecules (templates). Some of such MTPAs were developed to target RBD of coronaviruses preventing infection of targeted cells (89).

### Antiviral Vaccines

**Fig. 4:**
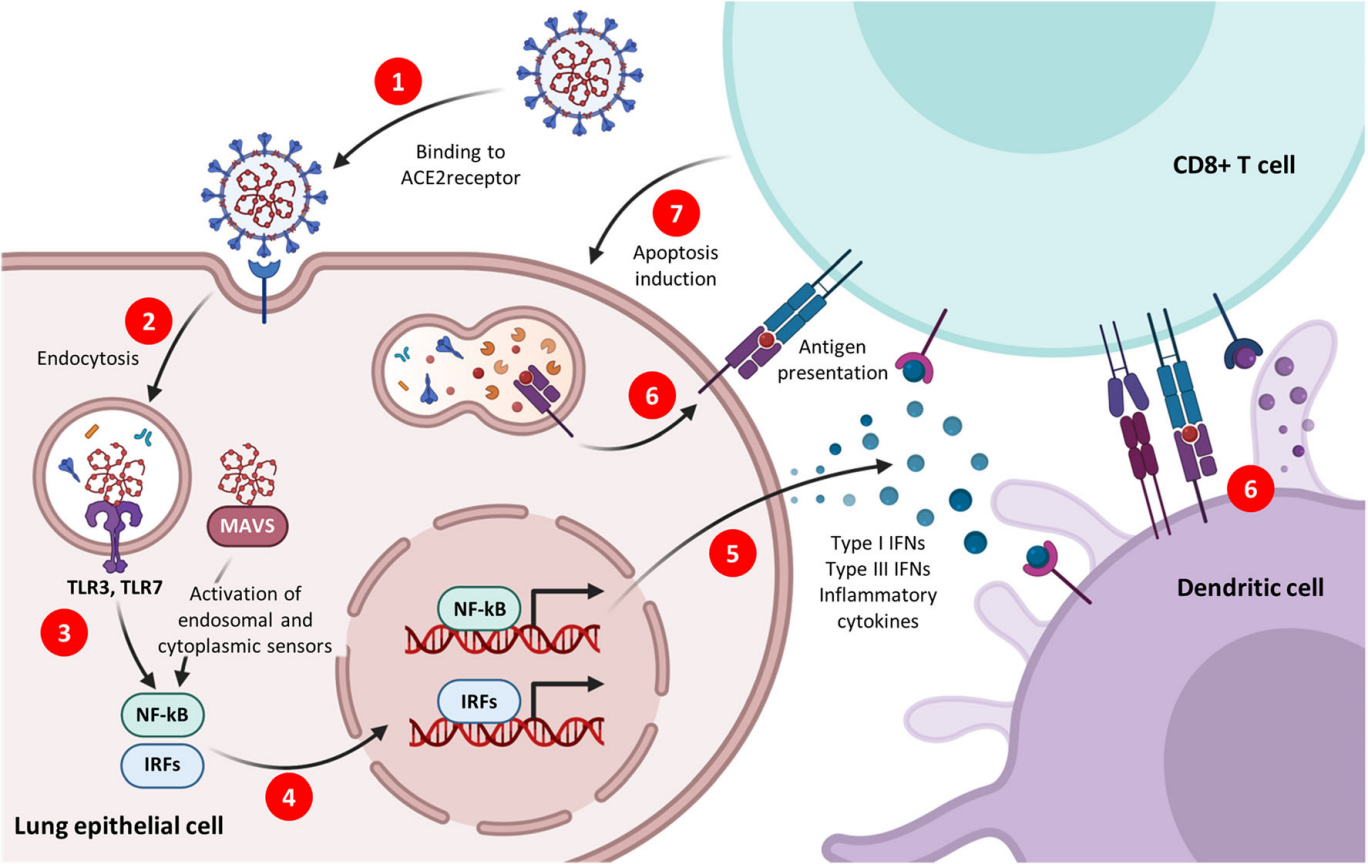
Acute Immune responses to coronaviruses. Coronaviruses are RNA viruses, some of which can infect human lung epithelium via the receptor ACE2 (1). After endocytosis (2), viral RNA activates endosomal and cytoplasmic sensors, TLR3/7 and MAVS, respectively (3). These receptors activate interferon regulatory factors (IRFs) and NFkB (4) to induce inflammatory cytokines (5), including interferons (IFNs). Dendritic cells (DCs) sample antigen and migrate to lymphoid organs to prime adaptive immunity. CD8 T cells recognize of antigen on DCs or infected cells (6) and induce apoptosis in affected lung epithelial cells (7)

Glycosylated spike protein is a major inducer of host immune response after infection of human lung epithelium cells via the receptor ACE2 (Fig. 4, step 1). After endocytosis (Fig. 4, step 2), viral RNA activates endosomal and cytoplasmic sensors, TLR3/7, and MAVS, respectively (Fig. 4, step 3). These receptors activate interferon regulatory factors (IRFs) and nuclear factor kappa B (NFkB) (Fig. 4, step 4) to induce inflammatory cytokines (Fig. 4, step 5), including interferons (IFNs). Dendritic cells (DCs) sample antigen and migrate to lymphoid organs to prime adaptive immunity. CD8 T cells recognize antigen on DCs or infected cells (Fig. 4, step 6) and induce apoptosis in affected lung epithelial cells (Fig. 4, step 7).

Vaccines are biological preparations that, when administered to an individual, stimulate the production of antibodies and provide immunity against one or several specific entities that can cause a disease, such as viruses or bacteria. Vaccines are prepared from the essential agent of a disease, its products, or a synthetic substitute, which are specifically prepared to act as an antigen without inducing the disease. Vaccines prime the immune system creating a form of memory allowing an individual to respond faster and with higher extent to a threat when compared with the first time response. Proteins, nucleic acids (DNA and RNA), or even entire organisms can be used for vaccination often in combination with adjuvants that can boost their potency. Vaccines, in contrast to most therapeutic drugs, are most commonly used for prophylactics of infection or disease. Therapeutic drugs directly affect the disease or, similar to vaccines, modulate the immune system increasing its power to fight the current incident of disease. However, therapeutic drugs usually provide no form of “memory” that can help the organism to fight against the repetition of similar infection or disease. From an individual point of view, the goal of vaccination is to prevent or modify disease. From a public health perspective, the goal of vaccination is to limit the spread of a pathogen within a population (90). If successful, personal defense and epidemic control lead to the complete elimination or extermination of the pathogen. Biological challenges including limiting efficacy, considerable variation in the immune response, antibody, and cellular immune marker levels among individuals, technical, operational, or logistical obstacles and social, ethical, religious, and political issues represent major factors that determine vaccine effectiveness (90).

**Fig. 5:**
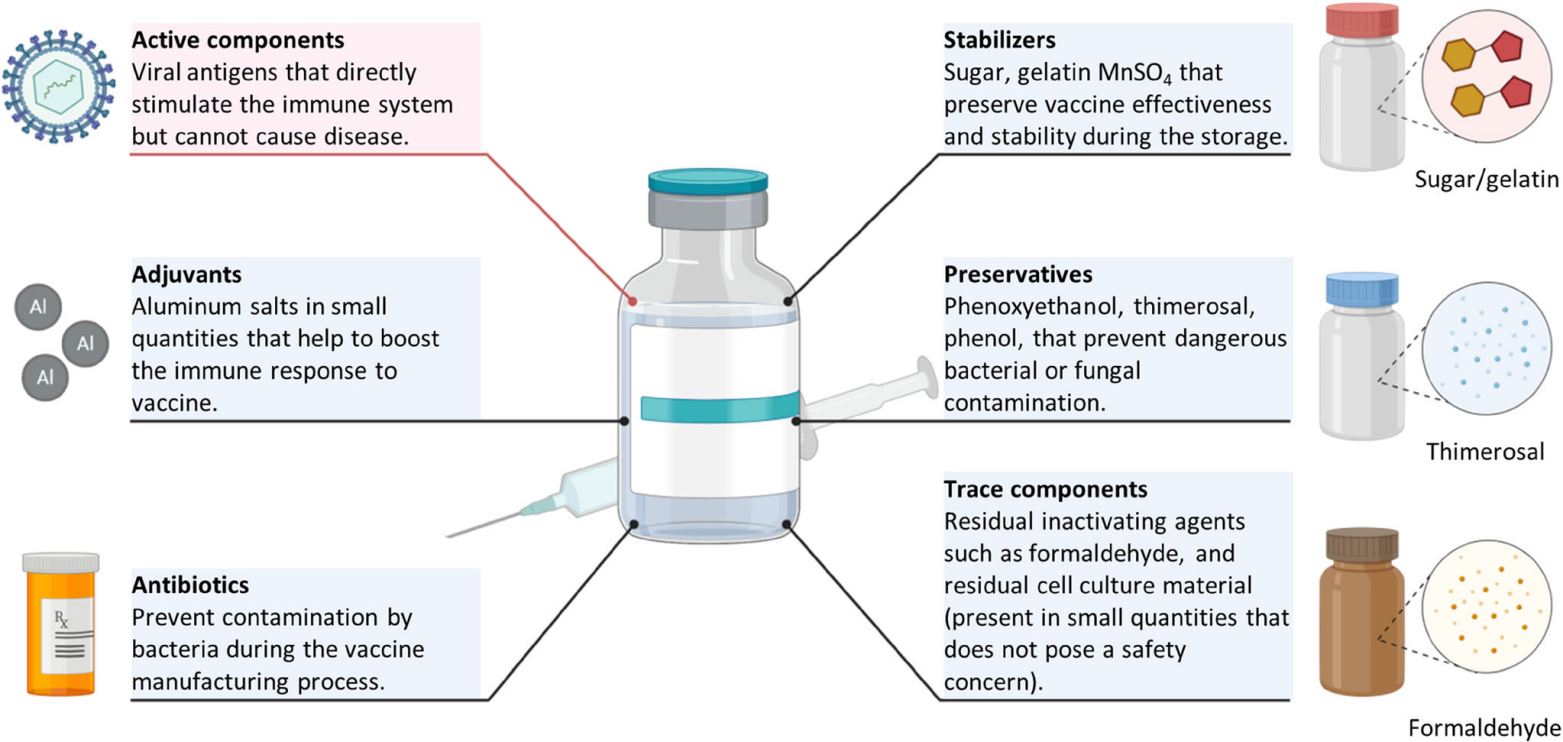
Common components of vaccines. Vaccines include various components such as active ingredients, adjuvants, antibiotics, stabilizers, preservatives, and traces of residual inactivating agents as schematically shown here

In general, vaccines contain several main components (Fig. 5). Antigen or active component is a major ingredient of a vaccine that induces an immune response against a disease or infection and triggers the development of “memory” in the immune system helping to fight against the next exposure to the threat. Antigens usually consist of altered forms of the virus, bacteria, toxin, and protein of genetic material that directly stimulate the immune system of an individual but do not cause the disease or produce weakened light symptoms of the illness. Each form of antigen has its own advantages and disadvantages (91). For instance, viral vectors (both replication-competent and replication-defective) usually induce good humoral and T cell responses and can be simply prepared from a virus culture but also may be reversed to virulence. Virus-like particles are usually safe and also induce good immune response. However, the cost of their production may be high and requires a relatively complex multistep procedure. Naked and encapsulated to nanoparticles nucleic acid and synthetic peptides are pretty safe but produce lower immune responses or uncertain immunogenicity linked to target populations. Inactivated viruses and self-disseminating wildlife vaccines are usually safe but demonstrate questionable (but sometimes effective) immunogenicity and may lead to the development of virulence (91).

Adjuvants are used for enhancing the immune response of the body. Aluminum salts are the most often used adjuvants. Precise mechanisms of action of adjuvants are still remaining unknown. Antibiotics are often used to prevent bacterial contamination of vaccines. Sugars, amino acids, and proteins are added to vaccines in order to protect them during the storage and preserve their efficiency. Preservatives are used to protect vaccines from bacterial or fungal contaminations after their manufacturing. The mercury-containing thiomersal is the most often used compound especially for vaccines that need to be injected several times from the same rubber-capped vessel. Phenol and phenoxyethanol are also used as vaccine preservatives. A number of trace components (e.g., formaldehyde used to inactivate viruses) are usually present in the vaccines in very low concentrations.

#### Repurposed Live Vaccines

Currently, 3 known repurposed vaccines are in phase 4 clinical trials: the oral polio, measles-mumps-rubella, and bacille Calmette-Guerin vaccines (36). The aim of phase IV clinical trials (also known as post-marketing surveillance) is at watching for the long-term effects of the drugs on a large population. Due to the longer term and larger subset of individuals using the therapeutic, extremely rare side effects can be observed.

The oral polio vaccine is an attenuated strain of the poliomyelitis virus (90). It is produced by the passage of the virus through non-human cells at low temperatures. Such passage produces random mutations in the virus and attenuates it. Studies have shown that this vaccine may possess non-specific actions, reducing morbidity and mortality under other infections. It is being tested to determine whether this vaccine will be effective against SARS-CoV-2 (92).

##### MMR

Measles, mumps, and rubella are viral diseases with serious consequences. The measles-mumps-rubella (MMR) vaccine consists of live-attenuated strains of these viruses and was shown to prevent these diseases (especially in children). It was suggested that MMR as well as possible other live vaccines may be useful in the prevention of other viral diseases including COVID-19 (93). One explanation of such effect suggests that antibodies formed in a response to the MMS or other live vaccines may be cross-reactive with SARS-CoV-2. Thus, vaccination with the MMR vaccine potentially may protect against COVID-19.

##### BCG

**Fig. 6:**
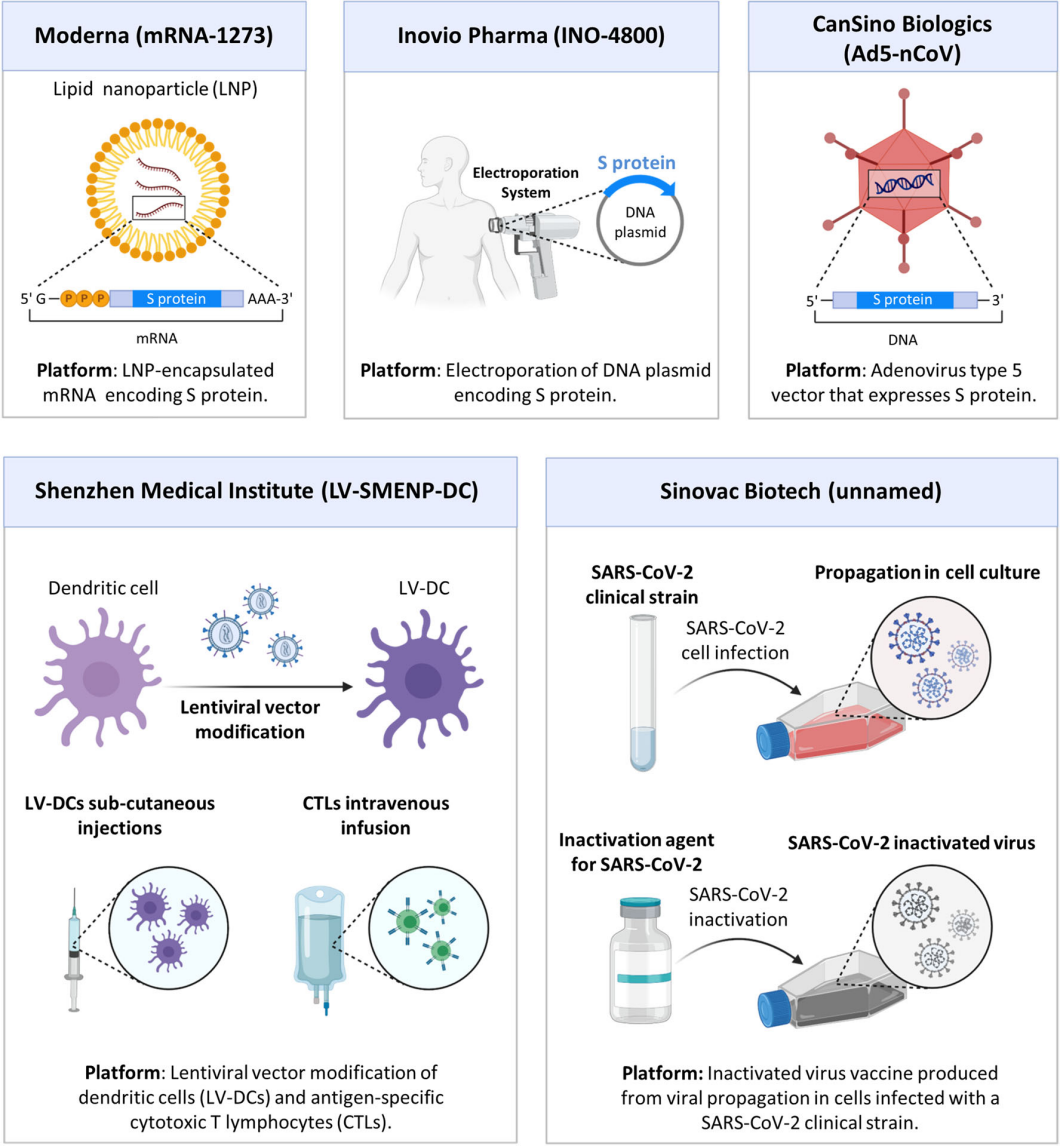
Clinical phase vaccine candidates for COVID-19 (as of April, 2020). Based on data from (36)

The bacille Calmette-Guerin vaccine is another live-attenuated vaccine consisting of the bacteria that causes bovine tuberculosis (*Mycobacterium bovis*). Remarkably, bacille Calmette-Guerin (BCG) vaccination seems to demonstrate non-specific protective effects against other respiratory tract infections (17). The analysis has shown that countries with high coverage of BCG have a low death rate from COVID-19 (94). Consequently, this vaccine was repurposed for the prevention of COVID-19 infections. Below, we are providing examples of several other vaccines of different types that currently are tested in clinical trials against coronaviruses (Fig. 6).

A common live vaccine (that protects against polio, MMR, BCG, etc.) could help in limiting the disease severity in patients with COVID-19. The first line of defense against any infection is the patient’s ability of own immune system to fight infection in a nonspecific way (i.e., innate immune response). The possible mechanism of action of these repurposed common vaccines against the SARS-CoV-2 infection may involve boosting patient’s own immune response. The repurposed common vaccines may benefit the immune system in a nonspecific way—thereby helping it to fight against the SARS-CoV-2 infection in an indirect way through innate immune response. Thus, repurposing common live vaccines might be a low-risk and high-reward strategy to save lives from the SARS-CoV-2 infection (93).

#### Clinical Phase Vaccine Candidates for COVID-19

Most of the vaccines are based on the injection of a genetic material encoding glycosylated spike (S) protein—a major inducer of host immune response. They differ in the type of nucleic acid used and the delivery approach. Moderna (mRNA-1273) represents an mRNA encoding S protein encapsulated into lipid nanoparticles (95). Preliminary data showed that the mRNA-1273 vaccine induced anti-SARS-CoV-2 immune responses in all participants, which granted further clinical trials. Two other vaccines are also based on the immunization with S protein, namely, Inovio Pharma (INO-4800) and CanSino Biologics (Ad5-nCoV) which use electroporation of a DNA plasmid encoding S protein and adenovirus type 5 vector that expresses S protein, respectively (96,97). These vaccines demonstrated promising results in experiments *in vivo* and *in vitro* and now entered clinical trials. Two other anti-COVID-19 vaccines were developed by the Shenzhen Medical Institute (LV-SMENP-DC) and Sinovac Biotech and are based on lentiviral vector modification of dendritic cells combined with antigen-specific cytotoxic T lymphocytes and inactivated SARS-CoV-2 clinical strain, respectively (36). The NVX-CoV2373 (clinical trial number NCT04368988) vaccine utilizes nanoparticles to deliver SARS-CoV-2 spike protein via intramuscular route. The purified full-length spike (S) proteins form small nanoparticles that are thermostable and bind with high affinity to the human ACE2 receptor. These nanoparticles are used in combination with a saponin-based Matrix M adjuvant which stimulate the entry of nanoparticles into the injection site and enhance antigen presentation in the local lymph nodes (36,98). COVID-19 vaccine candidates V591 (clinical trial number NCT04498247) and TMV-083 (clinical trial number NCT04497298) employ an attenuated measle virus as a vector to coronavirus proteins (36,99,100). LY3819253 virus candidate (clinical trial numbers NCT04411628, NCT04427501, NCT04497987, NCT04501978) represents an IgG1 monoclonal antibody against the COVID-19 spike protein. This protein was isolated from one of the first COVID-19 patients in North America (36).

### Convalescent Plasma Therapy

Convalescent plasma therapy is one of the investigational treatments for COVID-19 disease. Convalescent plasma therapy, in which the antiviral antibodies produced in the plasma from the recovered patient are injected in the patient, has shown promising results in various viral diseases such as H5N1 influenza and Ebola viral disease (EVD) (101–105). Convalescent plasma containing neutralizing antibodies against the SARS-CoV-2 virus is collected from the recovered patient and administered to the COVID-19 patients in order to increase the power of immune system or to boost patient’s immune response for the virus immediately after the infection. Shen *et al.* observed that convalescent plasma therapy improved the severe cases of COVID-19 (106,107). Clinical trial was also conducted in several countries to evaluate the recovery rate of COVID-19 patients after such a therapy (108). Convalescent plasma therapy could be a therapeutic option for the SARS-CoV-2 infection. As per the guidelines given by the US-FDA (United States-Food and Drug Administration), only COVID-19 survivors who were tested negative in nasopharyngeal swab test or molecular diagnostic test twice with no clinical symptoms at least 28 days from discharge can donate convalescent plasma to the COVID-19 patients (109). It should be however stressed, which based on the nature of convalescent treatment, it should be most effective in the early stage of infection before a significant damage of organs.

### Immunotherapy

While the induction of immune response is essential for fighting against the coronavirus infection, in some patients, disease progression leads to an enormous secretion of cytokines, known as cytokine storm (110). This hyperreaction often leads to the inflammation, lung damage, and finally may cause acute respiratory distress syndrome (ARDS) and even the development of pulmonary fibrosis. If that is the case, treatment of the coronavirus infection by antiviral therapy alone may not be sufficient to effectively fight viral infections and associated pathologic conditions. Consequently, in some cases, anti-inflammatory treatment or even the suppression of overactive immune response might be beneficial. The suppression of pro-inflammatory signaling pathways by monoclonal antibodies (tocilizumab, sirukumab, olokizumab, clazakizumab, etc.) as well as other inhibitors of inflammatory pathways (like baricitinib and ruxolitinib, which also inhibits clathrin-mediated endocytosis and therefore suppresses penetration of viruses into cells preventing infection) may be (and in some cases were) beneficiary on the advanced stages of COVID-19 (110–113). Dapsone, colchicine, and olanzapine were also suggested for the prevention of COVID-19-associated ARDS (114).

### Computer-Aided Design of Antiviral Therapeutics

Target-based drug design is a new strategy in modern drug discovery research (115,116). By exploiting computational tools, researchers have recently attempted to predict and design small molecule, peptide-based antiviral agents against various targets of SARS-CoV-2 viral disease. We have summarized a few of such computer-aided and target-based prediction and design of antiviral agents for COVID-19.

#### RdRp

RNA-dependent-RNA polymerase (RdRp) is an essential component for virus replication process (117). Therefore, inhibiting the function of RdRp could be beneficial for reducing the replication of SARS-CoV-2 virus. Recently, Zhang *et al.* reported binding interaction of three clinically used antiviral drugs as RdRp inhibitor, namely, galidesivir, favipiravir, and penciclovir which were also under clinical trial for COVID-19 disease. The interactome profile of these drugs with the RdRp protein provides information regarding the structure-based design of inhibitors for SARS-CoV-2 inhibition (118).

#### 3CLpro

The viral 3-chymotrypsin-like cysteine protease (3CLpro) enzyme which controls replication of the SARS-CoV virus is a potential target for an antiviral therapy (119). Because of similar genome sequence of SARS-CoV-2 and that of SARS-CoV, 3CLpro can be a target for SARS-CoV-2 virus. Recently, researchers have exploited the computer-aided drug design (CADD) approach to identify repurposing drug candidate for COVID-19. For example, Wang *et al.* performed virtual screening of a series of known drugs against the active site of key viral proteins of SARS-CoV-2 virus and identified several drugs including lopinavir, carfilzomib, elbasvir, valrubicin, and eravacycline as predicted inhibitors of SARS-CoV-2 protease enzyme. This technique can quickly predict repurposing drug candidates, and further design of novel inhibitors for SARS-CoV-2 protease can be directed based on the structure of these predicted drugs (120).

#### ACE2

It is known that SARS-CoV-2 virus is fused inside the host cell through angiotensin-converting enzyme II (ACE2) receptor, which was also found as host cell receptor for previous SARS-CoV virus (121). Receptor-binding domain (RBD) of spike (S) protein is a ligand on the surface of SARS-CoV-2 (corona) that binds to the ACE2 receptor facilitating the receptor-mediated endocytosis of the virus inside the host cell (Fig. 3a). The CR3022, a known SARS-CoV neutralizing monoclonal antibody with the receptor-binding domains (RBD) of the SARSCoV-2 spike protein can bind the SARS-CoV-2 spike protein (122,123). The crystal structure of “SARS-CoV-2 spike protein-ACE2” made it easier to design new therapeutics for ACE2 (124,125). In a recent work, Pentelute’s group have designed a new 23-mer peptide mimic which showed *K*_*d*_ value of 47 nM, against the S-spike protein and 7 nM for full-length ACE2 protein (126). Such computer-designed peptide-based therapeutic can be a promising candidate for the treatment of COVID-19 disease.

#### Endopeptidase of SARS-CoV-2 Virus

Lopinavir and/or ritonavir which are alone or in combination showed activity against the replication of human immunodeficiency virus (HIV) (127,128). In earlier studies, both of lopinavir and ritonavir and their combination treatment showed therapeutic benefits in patients with SARS-CoV (129,130). In a recent computational study, both lopinavir and ritonavir were found to bind with the endopeptidase of SARS-CoV-2 virus (131) indicating that these drugs might also show activity against the SARS-CoV-2 virus which has a similar feature with the SARS-CoV virus.

### Nanotechnology-Based Drug Delivery

#### Nanotherapeutics

Nanomaterial-based technologies have opened an emerging field in the past decade in drug delivery research, where nanosized materials and methods have been exploited for both disease diagnosis and therapeutics applications (132–135). In recent years, researchers have also explored various nanoscale materials for effective delivery of antiviral drugs (136,137). Nano-based drug delivery systems and methods have been evaluated for the previous coronavirus diseases SARS-CoV and MERS-CoV. For example, Coleman *et al.* prepared spike nanoparticles and tested them with combination of adjuvants in mice bearing SARS-CoV and MERS-CoV infection separately (138). The authors observed production of high titer antibodies in mice against these viral infections. These results indicated that the spike nanoparticles could neutralize the antibody response in mice. Such nano-based spike nanoparticle can be a promising option for nanovaccine against coronavirus infections. Huang *et al.* developed a gold nanorod system composed of a peptide inhibitor specific to heptad repeat 1 (HR1) receptor for the treatment of MERS-CoV disease (139). The authors reported 10-fold higher inhibition of HR1-/HR2-mediated membrane fusion of MERS-CoV virus in the host cells as compared with the inhibitor treatment alone. Such gold nanorod-based system can be effective in treating MERS-CoV and other coronavirus infections. In 2013, Li *et al.* demonstrated that RNA interference (RNAi) can mediate antiviral immunity in mammals (140). Since SARS-CoV, MERS-CoV, and SARS-CoV-2 coronaviruses are RNA viruses; therefore, small interfering RNA (siRNA) might be an effective therapy to inhibit the growth of such RNA viruses through silencing their viral gene activity. Currently, there is no nanoparticle-based therapeutics for the treatment of COVID-19; however, research and development of nanotherapeutics for COVID-19 are in progress. Nanomedicine approaches based on the reformulation of known antiviral drugs could be a promising therapeutic option for COVID-19 disease.

#### Inhalation Delivery for Treatment of ARDS

Treatment of virus-associated ARDS and the prevention of the development of lung inflammation and pulmonary fibrosis seems to be a valuable therapeutic approach that can supplement and enhance antiviral treatment and potentially improve the overall outcome. Furthermore, any treatment of lung injury should be performed via local intrapulmonary delivery of drugs. Inhalation or intratracheal delivery of anti-inflammatory drugs, antioxidants, hormones, or other biologically active substances specifically to the lungs or even only to the deceased cells has a potential to enhance the treatment of main lung injury, to the certain extent prevent drug penetration into the systemic circulation and therefore limit possible adverse side effects upon healthy organs and tissues (135,141–144). Previously, we proposed, developed, and tested on animal models inhalation and intratracheal treatment of severe hypoxia associated with lung edema, idiopathic pulmonary fibrosis, and lung manifestations of cystic fibrosis by nanocarrier-based therapeutics including alpha-tocopherol (145), prostaglandin E2 alone (146), or in combination with siRNAs for the suppression of inflammation and lung injury (147) and other drugs (148). We suggested that such treatment approaches may be also successfully used for prevention and treatment lung hypoxia, inflammation, and cystic and pulmonary fibrosis associated with COVID-19.

#### Combinatorial Nanotechnology-Based Treatment

Various latest therapeutic approaches such as broad-spectrum antiviral therapeutics instead of single target antiviral drug, combination therapy of antiviral drugs with antibiotics, and nano-encapsulated antiviral drugs and vaccines have displayed promising results for the treatment of COVID-19. Similarly, to other diseases, a combinational therapy of COVID-19 should be based on the simultaneous use of agents with different mechanisms of action (149). A combination of two or more drugs with significant activity and various therapeutic mechanisms potentially can provide a summary effect that is higher than just the arithmetical sum of their individual activities (drug synergy). Such synergy may be achieved by applying drugs targeting the same cellular or viral system (otherwise different parts of it) or by combining a drug which target a major disease (in our case the virus) and other active components that influence bioavailability, degradation, excretion, or resistance to the treatment. In addition, this multifunctional combinatorial treatment should be applied specifically to the targeted deceased cells leaving bystander healthy cells intact. Previously, we successfully applied this approach for the treatment of cancer by developing a targeted complex multifunctional proapoptotic anticancer drug delivery system by combining in one cancer-specific-targeted drug delivery system an inducer of cell death with suppressors of pump and non-pump resistance (142,150–152). Although, the targeting ligands for COVID-19 virus particles or infected lung cells are not discovered yet, targeting to the lungs as a primary site of action of the virus can be achieved by the local inhalation delivery of antiviral agent specifically to the lungs. Moreover, by using nanoparticle-based therapeutics, it is possible to combine in one complex delivery system antiviral drugs with drugs targeted to ARDS and therefore enhance the treatment of primary viral infections and secondary lung injury associated with COVID-19. Currently, we are testing this hypothesis in our laboratory.

## COVID-19: DIAGNOSTIC APPROACHES

Diagnostic tests are essential for monitoring and prognosis of every stage of a disease. Since the report of SARS-CoV-2 infection in December 2019, various assay kits and tests have been developed for the diagnosis of COVID-19. Current commercially available COVID-19 test is mostly dominant on the molecular genetic assays for detection of viral RNA from a clinical sample of SARS-CoV-2 infection using reverse transcription-polymerase chain reaction (RT-PCR) method. However, other techniques such as isothermal nucleic acid amplification assays, serological and immunological assays for anti-SARS-CoV-2 antibody, hybridization microarray assays, and chest CT scan are also promising for diagnosis of COVID-19 (153–155). Identification of the asymptomatic cases remains the main challenge to prevent the spread of SARS-CoV-2 infection. Therefore, development of accurate and rapid testing methods is required to prevent asymptomatic spread of SARS-CoV-2 infection. Researchers around the world have been developing various diagnostic methods because of current demand of cost-effective and point-of-contact test for the confirmation of SARS-CoV-2 infection. Here, we have summarized recent progresses on diagnostic methods for COVID-19 disease.

### PCR-Based Test

**Fig. 7:**
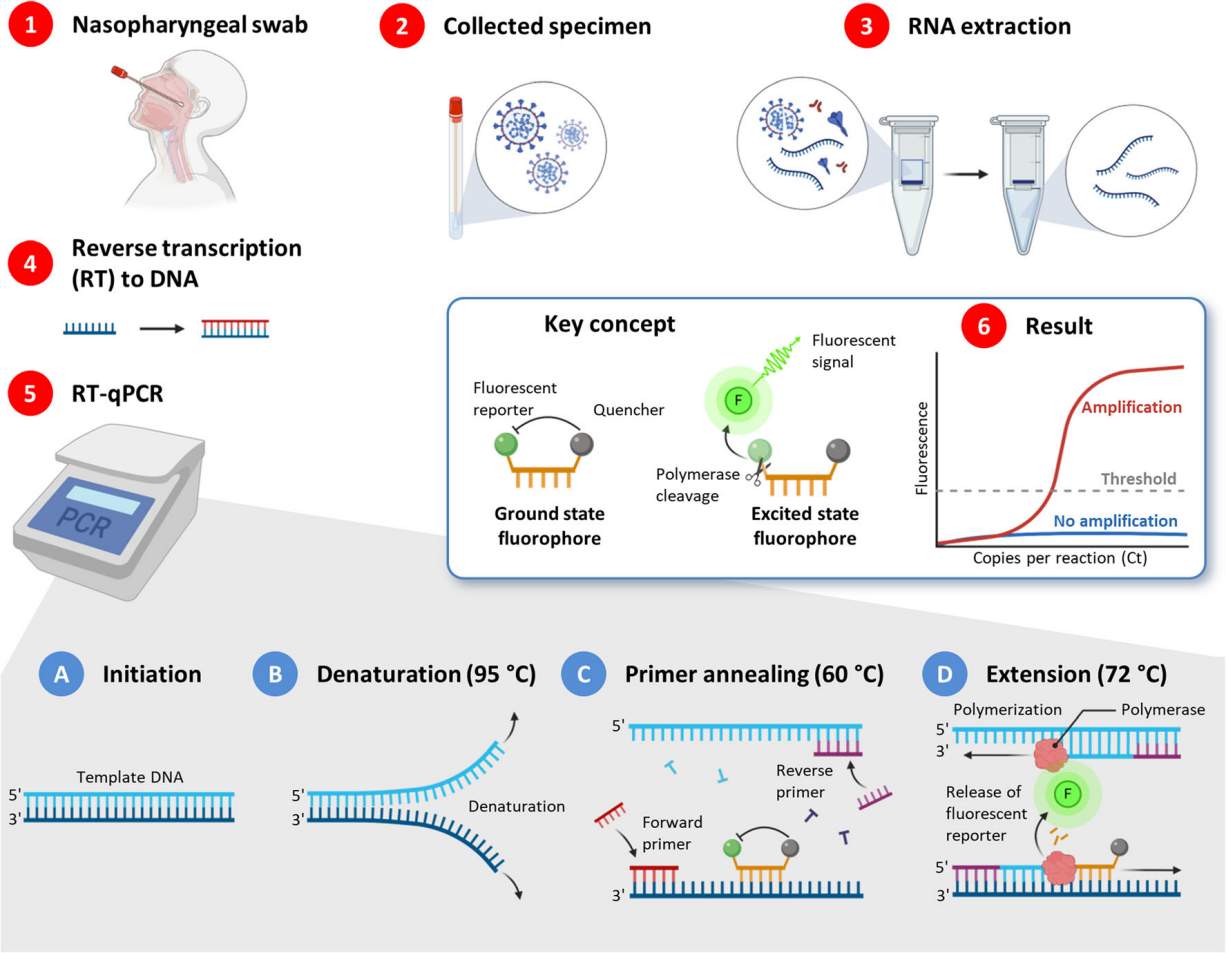
A typical procedure of COVID-19 diagnostics through RT-PCR. The RT-PCR method involves sample collection and extraction of viral RNA sample. The extracted RNA sample is converted to its complementary DNA (cDNA) by the reverse transcription. Finally, the amplification of this DNA sample is performed in qPCR, and the viral cDNA is detected by a fluorescent signal

Currently, the reverse transcription-polymerase chain reaction (RT-PCR) is the gold standard test for the diagnosis of COVID-19 (16). Mostly nasal swab and ocular secretions samples are being used in the TR-PCR test (156,157). Self-collected saliva sample was also explored by the researcher from the Rutgers Clinical Genomics Laboratory (158). Saliva sample collection is not only easy and quicker, but also, it has less risk to the healthcare personnel (159). Figure 7 shows stepwise procedure of the RT-PCR test for the detection of viral genome from the clinical sample. As shown in the figure, the RT-PCR test involves isolation of viral RNA from the clinical sample followed by generation of complementary DNA (cDNA) by RNA-dependent DNA polymerase reaction. Next, cDNA is converted to double-stranded DNA (dsDNA) through PCR amplification, and the amplification of this DNA sample is performed until the viral cDNA is detected by a fluorescent or electrical signal (160). The key concept of the fluorescence detection in quantitative fluorescence PCR is shown in the insert of Fig. 7. A substance marked with a fluorophore is added to the PCR mixture containing a DNA template, specific primer(s), deoxyribonucleotides, and a thermostable DNA polymerase in a suitable buffer solution. The fluorescence of the dye is suppressed by the quencher. After the cleavage of the fluorophore from a quencher by the polymerase, free dye emits fluorescence signal which is registered by a corresponding sensor in a thermal cycler. Each PCR cycle includes several required specific steps initiated by rapid changes in the temperature regimen. After initiation with a template DNA (Fig. 7, step A), the increase of temperature to 95°C causes the denaturation of double-stranded DNA leading to the separation of sense and antisense strands of the template (Fig. 7, step B). Rapid change of temperature to the most suitable value (usually 50–60°C) causes the annealing of primers and fluorophore-bound reporter probes to the corresponding strands of the template (Fig. 7, step C). Finally (Fig. 7, step D), polymerase extends both strands of the template and cleaves the fluorophore out of its quencher causing the emission of the signal. Each step leads to doubling template strands (or their short fragments after few initial steps). Totally, around 25–50 cycles are used allowing semi-quantitative or even quantitative (in number of copies) detection of the template DNA (in our case generated by coronavirus RNA during the reverse transcription). The RT-PCR test needs multiple temperature changes in each cycle, laborious instrumental work, and days to get the results. Therefore, there is an urgent need for rapid and cost-effective diagnostic methods, which can detect directly the presence of SARS-CoV-2 virus in clinical samples. Isothermal nucleic acid amplification which needs no change of temperature for each cycle is an easier technique for the detection of SARS-CoV-2 infection. Using constant temperature amplification method, Park *et al.* developed reverse transcription loop-mediated isothermal amplification (RT-LAMP) test for SARS-CoV-2 virus (161). Besides, researchers around the world are improving the sensitivity and timelines of the RT-PCR-based method. Seo *et al.* reported a field-effect transistor (FET)-based biosensor for quick detection of SARS-CoV-2 infection in patient samples. The main advantage of this highly sensitive biosensor device is that this method does not need any labeling of sample for the analysis. The authors prepared this biosensor by decorating the graphene sheets of the FET with an anti-SARS-CoV-2 antibody and evaluated its performance using cultured SARS-CoV-2 virus and swab specimens of COVID-19 patients. This EFT biosensor was capable of detecting SARS-CoV-2 spike protein at a very low concentration such as 1 fg/mL in phosphate-buffered saline, clinical samples, and cultured sample of SARS-CoV-2 virus—making it an extremely sensitive diagnostic method for SARS-CoV-2 infection (162). In another attempt, Wang *et al.* designed a novel one-step single-tube nested quantitative real-time PCR (OSN-qRT-PCR) method for the rapid detection of SARS-CoV-2 pathogen in clinical samples. The authors evaluated clinical performance of the OSN-qRT-PCR assay using several clinical samples and observed that this assay kit was capable of detecting SARS-CoV-2 virus in clinical sample with low viral load thereby making it more sensitive than the other qRT-PCR methods. This OSN-qRT-PCR assay could be a promising tool for the diagnosis of COVID-19 (163).

### Serological Test

**Fig. 8:**
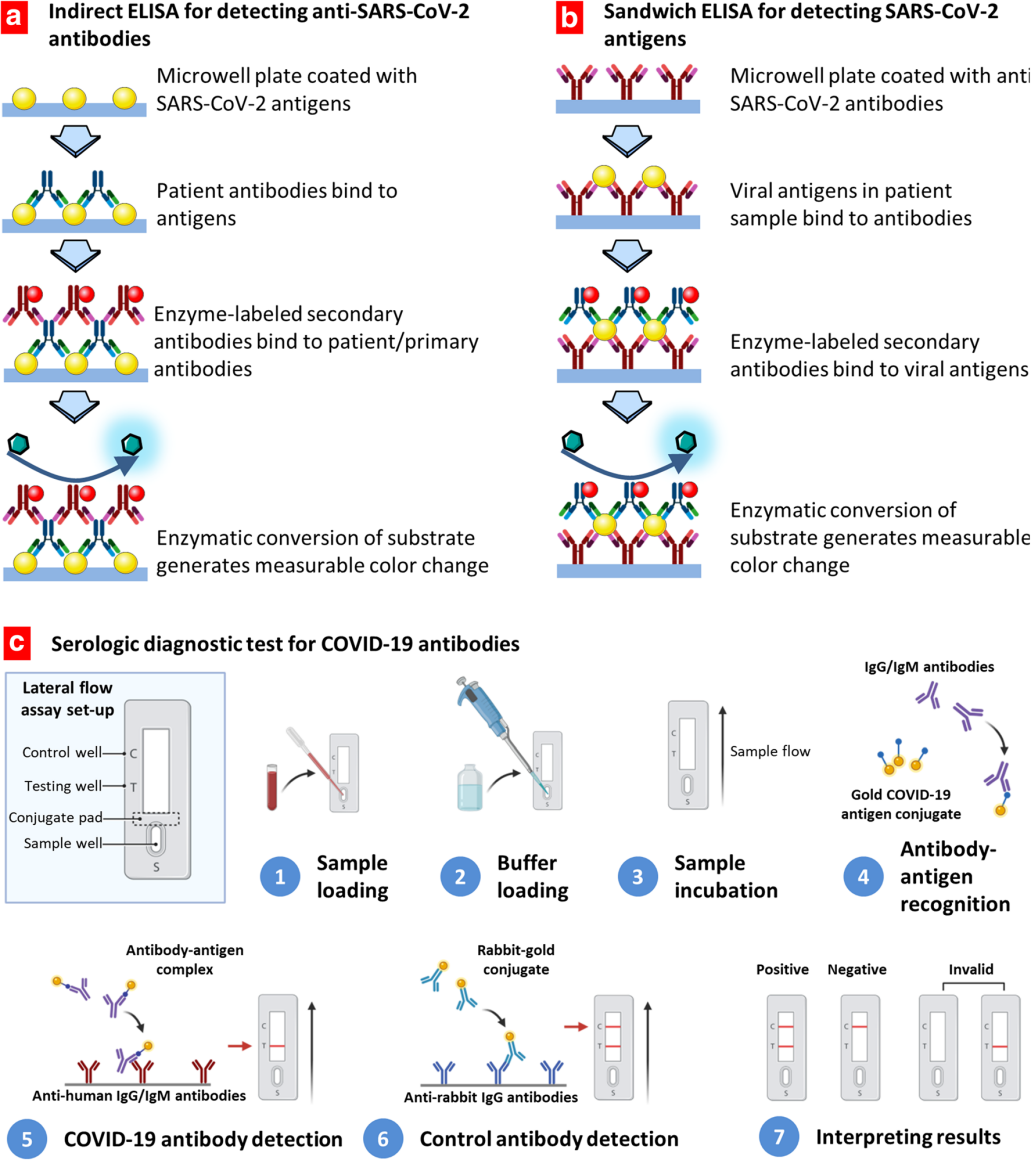
Assay techniques and tests for COVID-19 diagnosis. **a**, **b** The enzyme-linked immunosorbent assays (ELISA) detecting COVID-19 antibodies (**a**) or antigens (**b**). Redrawn with permission from (155). **c** Serologic diagnostic tests for COVID-19 antibodies

Serological test which involves analysis of blood serum or biological fluids to identify the presence of certain biomarkers such as antibody is generally used to monitor the progress of the disease. Various serological tests such as enzyme-linked immunosorbent assay (ELISA) have been explored to identify the people who have developed antibodies against SARS-CoV-2 virus infection (164,165). Recently, the FDA also authorized for a lateral flow serological immunoassay, namely, qSARS-CoV-2 IgG/IgM rapid test, manufactured by Cellex Inc. (166). A schematic illustration of ELISA-based serological tests is shown in Fig. 8. Two types of ELISA immunoassays are developed: detecting of COVID-19 antigens or antibodies against virus antigens. These assays are similar and differ in coating of a microwell plate. When it is coated with antigens, it is used for the detection of antibodies generated against the virus. Vice versa, coating with antivirus antibodies allows for the detection of the viral antigens. As shown in this figure, first, the patient sample containing the viral antigens is added to the anti-SARS-CoV-2 antibody-coated microwell plate, followed by addition of an enzyme-labeled detection antibody. At the end, a substrate suitable for the enzyme of the detection antibody is added to produce a colorimetric signal, which can be measured using a plate reader.

### Nanoparticle-Based Serological Tests

Nanoparticle-based colorimetric bioassays have been growing rapidly in recent years as a promising diagnosis tool for many diseases mainly due to simple instrumentations and visual output of the nano-based diagnostic tools (167). Because of localized surface plasmon resonance and inherent photostability properties, gold nanoparticles (AuNP)–based colorimetric bioassays have received significant attention in the development of image-based diagnostics (168,169). Recently, researchers have also explored AuNPs for possible diagnosis of COVID-19 disease. For example, Huang *et al.* prepared a gold nanoparticle–based lateral-flow (AuNP-LF) system made of various easily available inorganic nanomaterials (170). This AuNP-LF system can detect the IgM antibody developed in serum sample of a patient against the SARS-CoV-2 viral pathogen. The detection AuNP-LF strip was prepared by coating the SARS-CoV-2 nucleoprotein as antigen followed by conjugating antihuman IgM antibody. This AuNP-LF strip system could detect the SARS-CoV-2 virus in clinical samples within 15 min. Therefore, this AuNP-LF-based tool has shown a great promise for fast detection of COVID-19 disease. A general scheme of rapid serological assay for the detection of both IgG and IgM antibodies against COVID-19 virus is presented in Fig. 8c. A small blood sample is loaded into a test strip followed by the addition of the reaction buffer. A sample is incubated allowing the gold COVID-19 antigen conjugates to react with IgG-/IgM-labeled antibodies. Antirabbit antibodies conjugated with similar labeled gold nanoparticles are used for the detection of control antirabbit IgG antibodies. The test is considered positive when two lines (for COVID-19 and control antibodies) are visualized. If only the control line is detected, the result is defined as negative. When no control line is shown, the test is considered invalid. Moitra *et al.* prepared gold nanoparticle (AuNP)–based sensor for naked-eye detection of SARS-CoV-2 virus in the clinical samples (171). The authors coated the surface of the AuNP with thiol-conjugated antisense oligonucleotides (ASOs) specific for the nucleocapsid phosphoprotein of SARS-CoV-2 virus. This AuNP system was capable of detecting the presence of SARS-CoV-2 viral stain within 10 min. This naked-eye detection method includes clinical sample collection from COVID-19 patient followed by isolation of viral RNA, which is incubated with the antisense oligonucleotide (ASO)–capped gold nanoparticles (AuNPs) for 5 min. Next, RNase H is treated with the resulting viral composite of ASO-capped AuNPs for 5 min at 65°C producing a visual precipitate from the solution. Such AuNP-based system can be a very convenient and cost-effective tool for visual detection of SARS-CoV-2 virus. Various current diagnostic approaches for COVID-19 have been summarized in Table II. Thus, nanotechnology-based methods have potential in the development of cost-effective, fast, and point-of-care detection of both of SARS-CoV-2 virus and the related biomarkers associated with this viral infection.

**Table II:** A List of Current Diagnostic Approaches for COVID-19

Diagnostic method	Aim	Variants	References
Detection of the virus	To detect whether a person is currently infected with SARS-CoV-2 virus	RT-PCR-based detection	(172)
		RT-LAMP-based detection	(173)
		CRISPR-based detection	(174)
		ELISA for detecting SARS-CoV-2 antigens	(155)
Antibody tests	To detect whether a person had SARS-CoV-2 infection in the past	Serologic anti-SARS-CoV-2 Immunoglobulin M (IgM) and immunoglobulin G (IgG), detection	(165,175)
		Nanoparticle-based lateral-flow assay	(170)
		Indirect ELISA for detecting anti-SARS-CoV-2 antibodies	(155)
Imaging method	To detect ground-glass opacity spots in the lungs with a peripheral or posterior distribution as a sign of COVID-19	Chest X-ray imaging	(176)
		Chest CT scan	(177,178)
		Lung ultrasound imaging	(179)

### CT Imaging

**Fig. 9:**
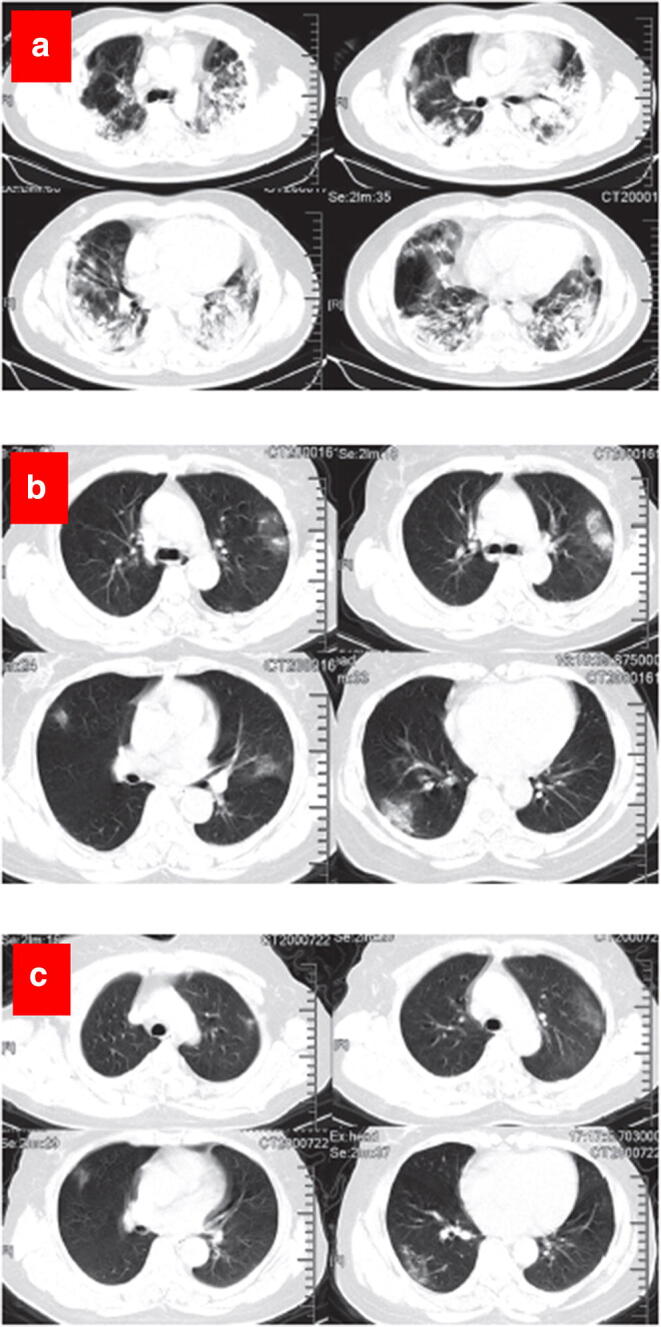
Representative chest CT images. **a** Transverse chest CT images from a 40-year-old man showing bilateral multiple lobular and subsegmental areas of consolidation on day 15 after symptom onset. **b** Transverse chest CT images from a 53-year-old woman showing bilateral ground-glass opacity and subsegmental areas of consolidation on day 8 after symptom onset. **c** Transverse chest CT images showing bilateral ground-glass opacity on day 12 after symptom onset. Reproduced with permission from (183)

Computed tomography (CT) scan was used as an early diagnosis tool for COVID-19 in many countries mostly due to lack of testing kits (180). Abnormal observations in the chest CT scan image were used as a diagnostic feature for COVID-19 (181,182). Usually, both bilateral and peripheral ground-glass opacities were observed in chest CT scans of COVID-19 patients in the early stage of the disease, whereas irregular-shaped paving patterns were observed at the later stage of the disease. Figure 9 shows chest CT images of ICU patients with COVID-19 in various stage of the disease. Figure 9a shows typical appearance of bilateral multiple lobular and subsegmental areas in the chest CT images. Figure 9b shows both bilateral ground-glass opacity and subsegmental areas in the chest CT images of non-ICU patients with COVID-19, and Fig. 9c shows only the bilateral ground-glass opacity in the chest CT images at later stage of the disease (183,184).

## CONCLUSIONS

The newly emerged COVID-19 viral disease caused by the SARS-CoV-2 pathogen has not only created severe panic among the people but also challenged the social culture and healthcare infrastructure all over the world. In this review, we have discussed briefly the cause of the COVID-19 disease, its symptoms, and management, and then, we have summarized recent progress on the development of various therapeutic and diagnostic approaches for the prevention, treatment, and diagnosis of COVID-19. We presented a diverse range of therapeutics such as antiviral therapeutics (repurposing of small molecule drugs), adjunctive therapeutics (neutralizing antibody, convalescent plasma treatment, etc.), nanotherapeutics (nanoparticle-based therapeutics and vaccines), and computational aided drug discovery (computation-based methods to predict and design new drug candidate) for COVID-19. Because of similar genome sequence of SARS-CoV-2 virus with the previous SARS-CoV virus, both the therapeutics and management methods for SARS-CoV infection have also been investigated for the COVID-19 disease. We have also summarized various diagnostic approaches developed for detecting and fighting COVID-19. Both researchers and clinicians around the world have been working for the development of vaccines as well as clinical trials of existing repurposed antiviral drugs against SARS-CoV-2 virus. While significant efforts have been made in recent time in the development of novel therapeutic and diagnostic approaches for COVID-19 disease, still many questions and challenges such as degree of sensitivity and specificity of serological tests for the detection of anti-SARS-CoV-2 antibody in clinical sample, whether the presence of such antibodies can produce immunity to this coronavirus etc., remain to be addressed. Although few antiviral therapeutics such as remdesivir, umifenovir, favipiravir, and ribavirin were evaluated in clinical trials and showed some positive results, still more evidences are needed for their successful clinical use against this disease. To date, there is no clinically approved therapeutics and vaccines for COVID-19. Therefore, early diagnosis such as testing of symptomatic and asymptomatic individuals and their close contacts by contact tracing, quarantine of the infected individuals, and effective supportive treatment of SARS-CoV-2-infected individuals are the key to prevent additional transmission and control this disease until any clinically approved antiviral therapeutics and/or vaccines for COVID-19 is available in the market.

## Abbreviations

3CLpro: 3-chymotrypsin-like cysteine protease
ACE2: angiotensin-converting enzyme 2
AOS: antisense oligonucleotides
ARDS: acute respiratory distress syndrome
AuNP: gold nanoparticles
AuNP-LF: gold nanoparticle-lateral-flow
BCG: bacille Calmette-Guerin
CADD: computer-aided drug design
cDNA: complementary DNA
COVID-19: coronavirus diseases 2019
CRISPR: clustered regularly interspaced short palindromic repeats
CT: computed tomography
ELISA: enzyme-linked immunosorbent assay
ER: endoplasmic reticulum
ERGIC: endoplasmic reticulum-Golgi intermediate compartment
EVD: Ebola viral disease
FDA: Food and Drug Administration
FET: field-effect transistor
HCQ: hydroxychloroquine
HIV: human immunodeficiency virus
HR1: heptad repeat 1
ICTV: International Committee on Taxonomy of Viruses
IFNs: interferons
IgG: immunoglobulin G
IgM: immunoglobulin M
IRFs: interferon regulatory factors
mAbs: monoclonal antibodies
MERS-CoV: Middle East respiratory syndrome coronavirus
MIPs: molecularly imprinted polymers
MMR: measles-mumps-rubella vaccine
MTPA: monoclonal-type plastic antibodies
OSN-qRT-PCR: one-step single-tube nested quantitative real-time PCR
PCR: polymerase chain reaction
RBD: receptor-binding domain
RdRp: RNA-dependent-RNA polymerase
RSV: respiratory syncytial virus
RT-LAMP: reverse transcription loop-mediated isothermal amplification
RT-PCR: reverse transcription-polymerase chain reaction
SARS-CoV: severe acute respiratory syndrome coronavirus
SARS-CoV-2: severe acute respiratory syndrome coronavirus 2
siRNA: small interfering RNA
ss-RNA: single-stranded RNA
TEM: transmission electron microscope
WHO: World Health Organization
